# Alb-PRF Hybrid Membranes Functionalized with Carbonated Hydroxyapatite and Doxycycline for Bone Regeneration and Antimicrobial Control: An In Vitro Study

**DOI:** 10.3390/ijms27083639

**Published:** 2026-04-19

**Authors:** Neilane Rodrigues Santiago Rocha, Emanuelle Stellet Lourenço, Victor Hugo de Souza Lima, Carlos Alberto Soriano, Alexandre Malta Rossi, Carolina N. Spiegel, Monica Diuana Calasans-Maia, Carlos Fernando Mourão, Gutemberg Gomes Alves

**Affiliations:** 1 Graduate Program in Science and Biotechnology, Fluminense Federal University, Niteroi 24210-201, Brazil; neilane_rocha@id.uff.br (N.R.S.R.); vh_souzalima@hotmail.com (V.H.d.S.L.); 2 Oral Surgery Department, Dental School, Fluminense Federal University, Rio de Janeiro 24020-140, Brazil; emanuelle_stellet@yahoo.com.br (E.S.L.); monicacalasansmaia@gmail.com (M.D.C.-M.); 3 Department of Condensed Matter, Applied Physics and Nanoscience, Brazilian Center for Research in Physics, Rio de Janeiro 22290-180, Brazil; sorianososo@gmail.com (C.A.S.); rossi@cbpf.br (A.M.R.); 4 Department of Cellular and Molecular Biology, Fluminense Federal University, Niteroi 24033-900, Brazil; carolinaspiegel@id.uff.br (C.N.S.); gutemberg_alves@id.uff.br (G.G.A.); 5 Department of Basic & Clinical Translational Sciences, Tufts University School of Dental Medicine, Boston, MA 02111, USA; 6 Clinical Research Unit, Antonio Pedro Hospital, Fluminense Federal University, Niteroi 24033-900, Brazil

**Keywords:** albumin-PRF, hydroxyapatite, doxycycline, bone regeneration, antimicrobial biomaterial

## Abstract

Bone tissue engineering requires biomaterials capable of simultaneously supporting regeneration and preventing infection. Platelet-rich fibrin (PRF) has been widely used due to its autologous origin and growth factor release, but its rapid resorption limits its clinical applications. Albumin-PRF (Alb-PRF) membranes were developed to improve stability, and their combination with carbonated nanostructured hydroxyapatite (nCHA) may further reinforce osteoconductive properties. In this proof-of-concept study, we fabricated Alb-PRF, Alb-nCHA-PRF, and Alb-nCHA-PRF + doxycycline (DOX) membranes and characterized their physicochemical, antimicrobial, and biological performance in vitro. Membrane stability was monitored for up to 14 days; DOX incorporation and release were evaluated by autofluorescence and spectrophotometry; antimicrobial activity was assessed against *E. faecalis* and *S. aureus*; and MG-63 osteoblast-like cells were used to test cytocompatibility, proliferation, mineralization, and alkaline phosphatase (ALP) activity. The release of 27 cytokines and growth factors was quantified by multiplex immunoassay. Alb-PRF exhibited morphological integrity and an enhanced trophic secretome, and supported proliferation and late mineralization. nCHA incorporation reduced cell proliferation and secretome output, while DOX conferred sustained antibacterial activity and enhanced early ALP expression even with attenuated cytokine release, positively impacting mineralization, when compared to nCHA alone. These preliminary results provide preliminary feasibility evidence that Alb-PRF can be engineered as a multifunctional scaffold combining antimicrobial and regenerative functions, though some trade-offs indicate the need for dose optimization and validation with in vivo models.

## 1. Introduction

Bone defects, commonly resulting from severe trauma, tumor resection, or chronic infection, present a significant clinical challenge in reconstructive surgery. While autologous bone grafting remains the clinical gold standard due to its inherent osteogenic, osteoinductive, and osteoconductive properties, this approach is constrained by significant limitations, including donor site morbidity, limited graft availability, and increased surgical complexity and time [1]. Up to 19% of bone grafts may fail to achieve complete consolidation, especially in large defects or poorly vascularized regions, as indicated by recent clinical analyses [2]. These drawbacks have created a compelling need for the development of advanced, readily available, and less invasive biomaterials that can effectively guide and promote endogenous bone regeneration.

Autologous platelet concentrates, particularly platelet-rich fibrin (PRF), have emerged as clinically relevant tools in regenerative medicine [3] due to their autologous origin and capacity to release growth factors that promote healing. PRF is a purely autologous, biocompatible fibrin matrix [4] which physically entraps a high concentration of platelets, leukocytes, and cytokines, functioning as a natural reservoir for regenerative mediators, and promoting cell migration, proliferation, differentiation, and angiogenesis [5]. However, conventional PRF membranes resorb rapidly and provide limited mechanical stability, which restricts their use as long-term scaffolds or barriers [6]. To overcome this limitation, a novel formulation known as albumin-PRF (Alb-PRF) was developed by combining liquid PRF with heat-denatured serum albumin [7,8]. This process creates a stable, biocompatible, and fully autologous fibrin–albumin hydrogel with markedly improved structural integrity and an extended resorption profile, remaining stable in vivo for up to four to six months [9,10]. Although the Alb-PRF formulation significantly improves stability and longevity, its preparation involves controlled heating and rapid handling, which may limit its standardization and scalability. Nevertheless, its enhanced longevity transforms the PRF-based scaffold from a short-term biological dressing into a versatile autologous platform suitable for further functionalization to meet the multifaceted demands of bone regeneration.

Further strategies to enhance the biological activity of Alb-PRF include its combination with inorganic phases such as hydroxyapatite (HA), a well-established calcium phosphate ceramic with recognized osteoconductive and osteointegrative properties [11]. Carbonated nanostructured HA (nCHA) is particularly compelling due to its superior biomimetic properties; the incorporation of carbonate ions into the apatite lattice more closely mimics the chemical composition of native bone mineral, which may enhance biological recognition and integration [12]. Recently, it was shown that the association of Alb-PRF with nCHA microspheres resulted in biocompatible composites able to stimulate osteoblast proliferation, differentiation, and in vitro mineralization compared with Alb-PRF alone [13], and a randomized trial evaluating nCHA spheres combined with blood-derived growth factors for maxillary sinus floor elevation demonstrated improved handling of the graft material [14]. It is important to note that combining inorganic mineral phases with protein-rich matrices can alter the distribution or adsorption of growth factors, potentially impacting the biological signaling of the composite.

Another critical challenge in bone regeneration procedures is the high risk of local infection, which can impair healing and compromise the outcome of bone grafting or implant therapy [15]. Postoperative infections remain a significant complication in bone reconstructive procedures, particularly in contaminated or compromised sites. Reported rates of surgical site infection (SSI) range from approximately 8% to 16% in oncologic or complex reconstructions involving large bone defects or prosthetic devices [2]. Systemic antibiotic administration often fails to achieve therapeutic concentrations at the poorly vascularized graft site and contributes to the global burden of antimicrobial resistance. Consequently, local antibiotic delivery systems integrated into regenerative biomaterials have been proposed as a superior strategy to provide high, sustained drug concentrations directly at the target site, minimizing systemic exposure [16]. Doxycycline, a broad-spectrum tetracycline antibiotic, is an ideal candidate for such a system due to its unique dual functionality. In addition to its established antimicrobial activity against common oral and orthopedic pathogens like *Staphylococcus aureus*, doxycycline possesses significant non-antimicrobial, host-modulatory properties that are highly beneficial for tissue regeneration, and has also demonstrated a role as a modulator of osteogenesis [17,18]. Prolonged or subinhibitory antibiotic exposure in local delivery systems can contribute to the development of resistance, emphasizing the need for careful dose optimization and release control.

These properties suggest that a hybrid biomaterial may combine regenerative and antimicrobial properties in a single autologous platform. In this context, we hypothesized that Alb-PRF membranes functionalized with nCHA and doxycycline would maintain cytocompatibility, support osteogenic responses, and provide local antimicrobial activity, while also modulating the release of cytokines and growth factors. Considering these interrelated biological and physicochemical variables, the present work was designed as a proof-of-concept in vitro investigation to explore the feasibility and early biological responses of this hybrid system. To verify this hypothesis, we developed and characterized this composite in vitro, evaluating its structural stability, drug release profile, antimicrobial effects, and biological responses in human osteoblast-like cells. The results provide preliminary evidence suggesting the potential of this strategy as a dual-function biomaterial for bone regenerative therapy.

## 2. Results

### 2.1. Preparation and Stability of Alb-PRF, Alb-nCHA-PRF and Alb-nCHA-PRF + DOX Membranes

Alb-PRF membranes were successfully obtained by combining heat-denatured albumin with liquid PRF, resulting in stable fibrin–albumin structures. The incorporation of carbonated nanostructured hydroxyapatite (nCHA) microspheres produced Alb-nCHA-PRF membranes, while pre-incubation of the microspheres with doxycycline yielded Alb-nCHA-PRF + DOX membranes. Alb-nCHA-PRF + Doxycycline membranes exhibited greater fragility, with partial detachment of microspheres evident after 7 days, while residual particles preserved their morphology.

**Figure 1:**
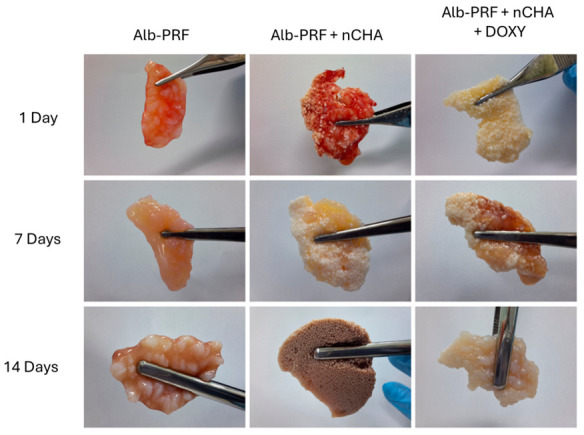
Macroscopic appearance of Alb-PRF, Alb-nCHA-PRF, and Alb-nCHA-PRF + Doxycycline membranes after incubation in culture medium for 1, 7, and 14 days.

During incubation in culture medium, Alb-PRF and Alb-nCHA-PRF membranes maintained their overall macroscopic integrity up to 14 days, with only minor contraction of the matrix. In contrast, Alb-nCHA-PRF + DOX membranes showed increased fragility, with progressive detachment of nCHA microspheres after 7 days, although the remaining particles preserved their original morphology. The incorporation of 1 mL of doxycycline solution into the Alb-nCHA-PRF formulation did not alter the apparent gelation time or gross morphology compared with the other membrane types. Representative images illustrating the preparation and stability of the three types of membranes are shown in Figure 1.

### 2.2. Assessment of Doxycycline Incorporation

**Figure 2:**
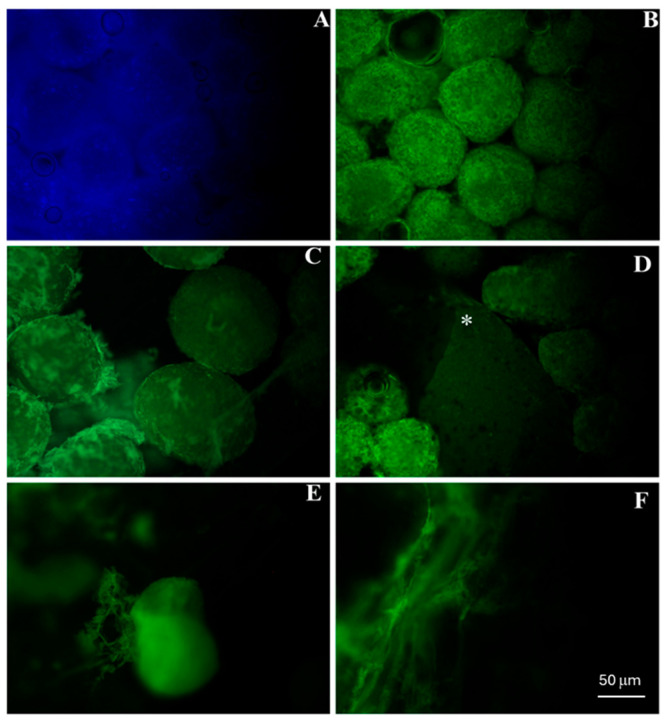
Autofluorescence of doxycycline in Alb-nCHA-PRF + DOX membranes after 7 days of culture. (**A**) Negative control (Alb-nCHA-PRF, no doxycycline), showing absence of fluorescence. (**B**,**C**) Strong green autofluorescence localized predominantly in nCHA microspheres. (**D**, asterisk) Fluorescence signal also detected in albumin gel. (**E**,**F**) Weaker distribution observed in the fibrin network. Images obtained under excitation at 405 nm; emission recorded at ~529 nm.

The incorporation of doxycycline into Alb-nCHA-PRF membranes was confirmed by fluorescence microscopy, taking advantage of the intrinsic autofluorescence of tetracyclines. Representative images of doxycycline autofluorescence in Alb-nCHA-PRF + DOX membranes compared with the negative control (Alb-nCHA-PRF) after 7 days of incubation are shown in Figure 2. A strong green fluorescence was detected predominantly within the nCHA microspheres, while weaker but detectable signals were also observed in the albumin gel and fibrin network, indicating partial distribution of the drug throughout the organic phase. At 14 days, fluorescence persisted mainly within the mineral phase, whereas no signal was detected in the albumin or fibrin components, suggesting preferential retention of doxycycline in the nCHA microspheres (Supplementary Figure S1).

### 2.3. Blood Cell Survival Within Membranes (Live/Dead Assay)

**Figure 3:**
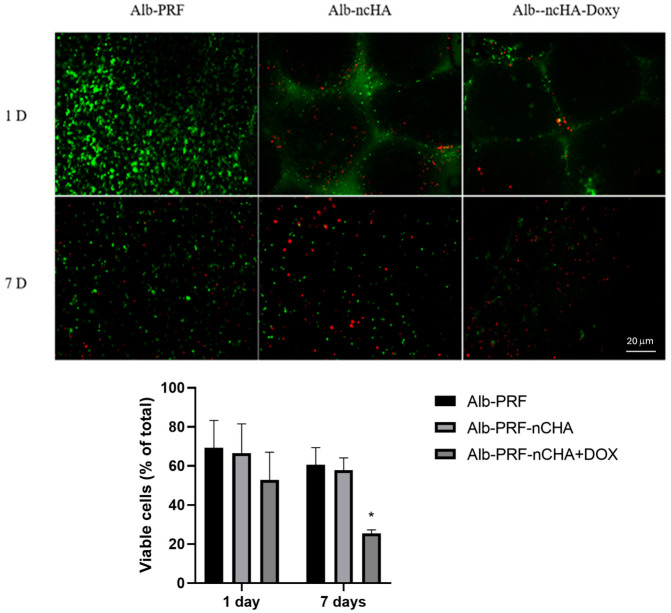
Cell viability assay (Live/Dead) of blood cells contained within Alb-PRF, Alb-ncHA-PRF (control), or Alb-ncHA-PRF + DOX membranes after 1 and 7 days of culture in DMEM. Viable cells appear green (calcein-AM) and non-viable cells appear red (EthD-1). Images were acquired by fluorescence microscopy, at 50× magnification. The lower panel shows quantitative analysis of cell viability. Data are presented as mean ± SEM of three independent biological experiments (*n* = 3 donors), each performed in five technical replicates (independent microscopic fields), which were averaged prior to analysis to generate a single value per biological replicate. An asterisk indicates significant differences between groups at the same experimental time (*p* < 0.05).

Fluorescence microscopy revealed distinct patterns of cell distribution and viability among the membrane types (Figure 3). Alb-PRF membranes exhibited a dense population of calcein-positive (green) cells at day 1, with a moderate reduction after 7 days, reflecting the natural remodeling of the fibrin–albumin matrix. In Alb-nCHA-PRF, viable cells remained visible along the fibrin network, but areas adjacent to the mineral microspheres showed more frequent EthD-1–positive (red) signals, suggesting localized stress or diffusion gradients. The Alb-nCHA-PRF + DOX membranes displayed a similar pattern, with a modest but consistent decrease in the proportion of viable cells over time. Quantitative analysis confirmed this trend: viability decreased gradually from day 1 to day 7 in all groups, more markedly in the presence of nCHA and DOX. These observations likely reflect the combined influence of sustained drug release and oxygen/nutrient diffusion limitations within the dense fibrin structure.

### 2.4. Drug Release Profile

Alb-nCHA-PRF + DOX membranes demonstrated a controlled and sustained release of doxycycline over the 7-day experimental period. During the first 96 h, the release profile remained relatively stable, followed by a significant increase at 168 h, when a peak concentration of 82.07 ± 8.46 µg/mL was detected (Table 1). This corresponded to approximately 0.364 mg of doxycycline, equivalent to 8.57% of the total drug incorporated per membrane (Table 1). These results suggest that the proposed delivery system is capable of providing a slow and sustained local release of doxycycline, with a delayed but consistent increase in drug availability at later time points.

### 2.5. Assessment of Antimicrobial Activity

**Figure 4:**
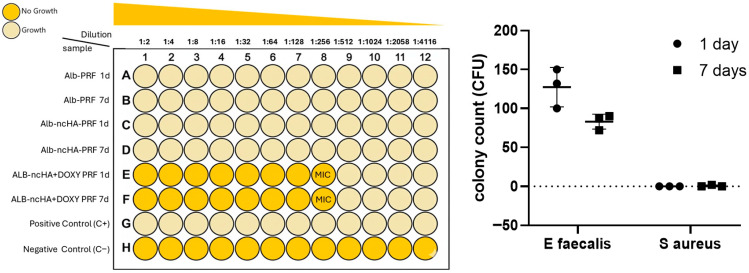
Antimicrobial activity of Alb-nCHA-PRF + DOX membranes against Enterococcus faecalis (ATCC 29212) and Staphylococcus aureus (ATCC 25923). Results of the broth microdilution assay are shown for eluates obtained after 1 and 7 days of membrane incubation. Wells with yellow shading indicate bacterial growth, whereas beige wells denote inhibition. The minimum inhibitory concentration (MIC) corresponded to the 1/256 dilution for both time points and bacterial species. The right panel shows quantitative colony counts from blood-agar subcultures at 1 and 7 days for both E. faecalis and S. aureus. Data are presented as mean ± SEM of three independent biological experiments (*n* = 3 membranes), each performed in 5 technical replicates (independent wells), which were averaged prior to analysis to generate a single value per biological replicate.

The antimicrobial effect of the different membranes was evaluated against *Enterococcus faecalis* (ATCC 29212) and *Staphylococcus aureus* (ATCC 25923) using the broth microdilution method. Eluates obtained from Alb-PRF and Alb-nCHA-PRF membranes did not inhibit bacterial growth at either 1 or 7 days. In contrast, Alb-nCHA-PRF + DOX eluates produced a pronounced inhibitory effect in both species. After 1 day, the minimum inhibitory concentration (MIC) was observed at the 1/256 dilution, corresponding to doxycycline concentrations between 0.16 and 0.32 µg/mL. The antimicrobial activity persisted in eluates obtained after 7 days, maintaining the same MIC level for both *E. faecalis* and *S. aureus* (Figure 4).

To distinguish bacteriostatic from bactericidal effects, aliquots from MIC wells were plated on blood agar, and colonies were quantified as colony-forming units (CFUs). For *E. faecalis*, CFU counts were reduced after 7 days compared with 1 day, confirming a sustained but predominantly bacteriostatic effect of doxycycline. In contrast, *S. aureus* exhibited no detectable colony growth, indicating complete bacterial eradication within the sensitivity limits of the assay and suggesting a bactericidal effect. These findings demonstrate that Alb-nCHA-PRF + DOX membranes provided persistent antimicrobial activity against both Gram-positive pathogens, with higher efficacy toward *S. aureus* (Figure 4).

### 2.6. Cytocompatibility Assays

**Figure 5:**
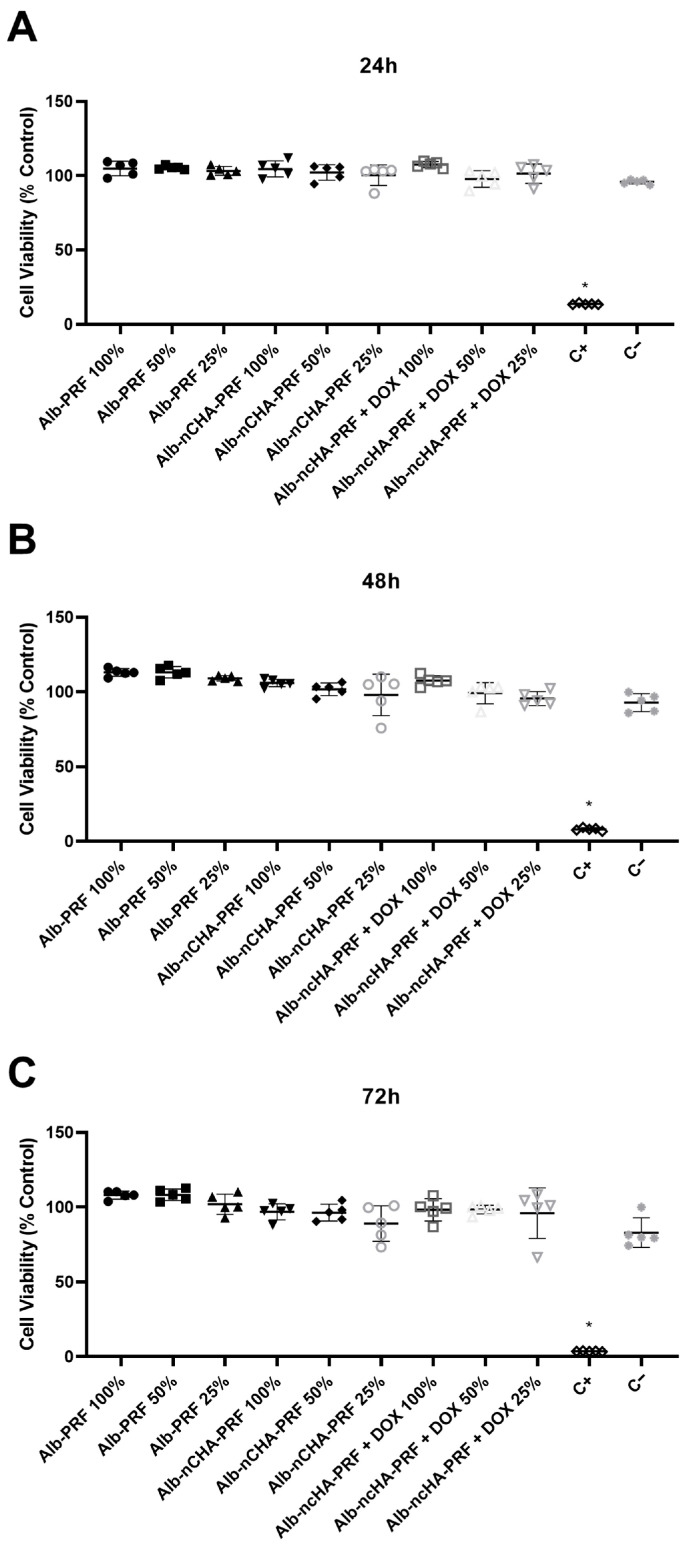
Cytocompatibility of membrane extracts prepared according to ISO 10993-12:2021. (**A**–**C**) Cell viability after 24 h (**A**), 48 h (**B**), or 72 h (**C**) of exposure to 100%, 50%, or 25% membrane extracts. Extracts were obtained by incubating each membrane in DMEM (serum-free, antibiotic-free) at 200 mg/mL for 24 h at 37 °C. Cell viability was quantified by the MTT assay and expressed as a percentage of the unexposed experimental control. Polystyrene beads were employed as a negative control (C−), and latex extract (2 mg/mL) was used as the positive control (C+). Data are presented as mean ± SEM of three independent biological experiments (*n* = 3 membranes), each performed in quintuplicate technical replicates (wells), which were averaged prior to analysis to generate a single value per biological replicate. * *p* < 0.05 versus all other groups.

Cell viability following exposure to membrane extracts is shown in Figure 5A–C. Across all extract concentrations (100%, 50%, and 25%) and timepoints (24, 48, and 72 h), none of the membrane groups exhibited a reduction in viability below the 70% threshold established by ISO 10993-5: 2009; Biological evaluation of medical devices—Part 5: Tests for in vitro cytotoxicity. International Organization for Standardization: Geneva, Switzerland, 2009, indicating absence of cytotoxicity. At 24 h (Figure 5A), viability values for Alb-PRF, Alb-nCHA-PRF, and Alb-nCHA-PRF + DOX remained close to the unexposed negative control, with no statistically significant differences among extract concentrations. Similar patterns were observed at 48 h (Figure 5B) and 72 h (Figure 5C), with viability consistently maintained within the 90–110% range for all membrane types.

As expected, the positive control (C+, extract of latex fragments) produced a marked decrease in cell viability at all points (*p* < 0.05), showing statistically significant differences relative to every other experimental group. The negative control (C−) remained stable throughout the experiment. Collectively, these results confirm that all membrane extracts are non-cytotoxic, even at the highest concentration recommended by the international standard (100% extract, equivalent to 200 mg/mL extraction conditions). Supplementary Figure S3 shows micrographs of all experimental groups, evidencing marked alterations in cell density and morphology exclusively in the positive control.

**Figure 6:**
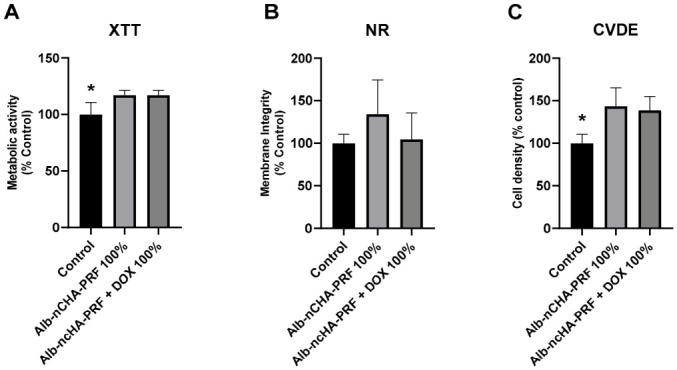
Multiparametric cytotoxicity assay in MG-63 cells exposed to 24 h eluates equivalent to 100% ISO 10993-12 extracts. (**A**–**C**) Alb-nCHA-PRF or Alb-nCHA-PRF+DOX groups assessed by XTT, neutral red (NR) uptake, and crystal violet dye exclusion (CVDE), respectively. Data are presented as mean ± SEM of three independent biological experiments (*n* = 3 membranes), each performed in quintuplicate technical replicates (wells), which were averaged prior to analysis to generate a single value per biological replicate. * *p* < 0.05.

The multiparametric cytocompatibility assay was performed using 24 h eluates obtained from Alb-nCHA-PRF and Alb-nCHA-PRF + DOX membranes and evaluated in MG-63 cells through three complementary endpoints: mitochondrial metabolic activity (XTT), lysosomal integrity (Neutral Red, NR), and cell density (Crystal Violet Dye Exclusion, CVDE). All conditions maintained cell viability above the 70% cutoff established by ISO 10993-5, confirming the absence of cytotoxic effects. In the XTT assay (Figure 6A), Alb-nCHA-PRF + DOX eluates induced a significant increase in mitochondrial activity compared with the medium control (* *p* < 0.05), whereas Alb-nCHA-PRF eluates showed values comparable to the control. NR uptake (Figure 6B) remained similar among all groups, with no statistically significant differences. In the CVDE assay (Figure 6C), Alb-nCHA-PRF + DOX also produced a significant increase in cell density relative to the control (* *p* < 0.05). Altogether, all eluates preserved cytocompatibility across the three parameters.

### 2.7. Assessment of Cell Proliferation

**Figure 7:**
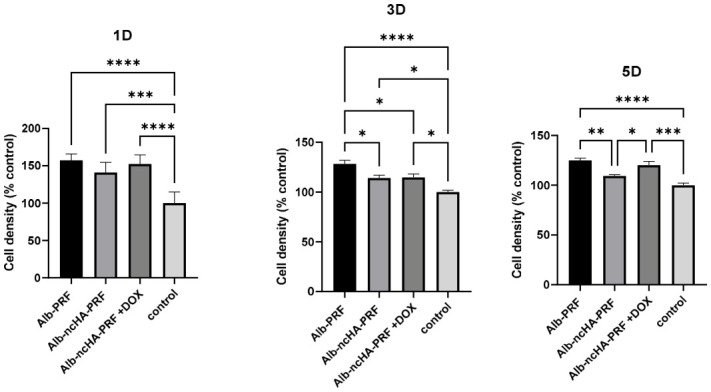
Cell proliferation of MG-63 osteoblast-like cells exposed to 25% eluates from Alb-PRF, Alb-nCHA-PRF, and Alb-nCHA-PRF+DOX membranes, evaluated by crystal violet dye exclusion (CVDE) assay at 1, 3, and 5 days. Data are presented as mean ± SEM of three independent biological experiments (*n* = 3 membranes), each performed in quintuplicate technical replicates (wells), which were averaged prior to analysis to generate a single value per biological replicate. Statistical analysis was performed using the Kruskal–Wallis test followed by Dunn’s multiple comparisons post hoc test. * *p* < 0.05, ** *p* < 0.01, *** *p* < 0.001, **** *p* < 0.0001.

The effect of membrane eluates on MG-63 proliferation was monitored over 7 days using the CVDE assay (Figure 7). At day 1, no significant differences were observed among groups, and all conditions showed similar cell numbers compared with the medium control. From day 3 onwards, Alb-PRF eluates promoted higher proliferation rates, with significant increases on days 3 and 5 compared with both Alb-nCHA-PRF and Alb-nCHA-PRF + DOX. At day 7, cell proliferation decreased in all groups, consistent with the attainment of confluence, although Alb-PRF still maintained slightly higher values than the other experimental conditions. Interestingly, the addition of doxycycline appeared to mitigate the reduction in proliferation associated with nCHA alone, as Alb-nCHA-PRF + DOX values were intermediate between Alb-PRF and Alb-nCHA-PRF (Figure 7).

### 2.8. Biomineralization In Vitro

**Figure 8:**
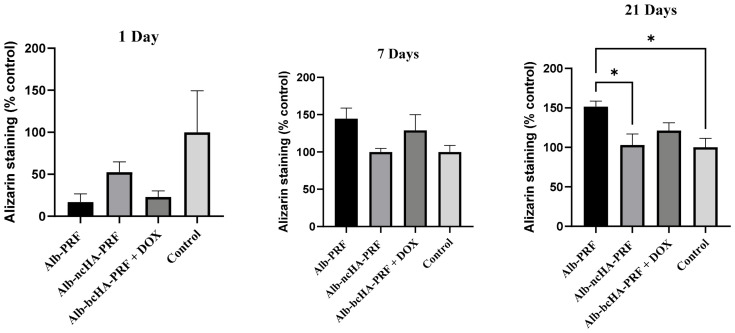
Calcium deposition in MG-63 cells exposed to eluates from Alb-PRF, Alb-nCHA-PRF, and Alb-nCHA-PRF+DOX membranes, evaluated by Alizarin Red staining at 1, 7, and 21 days. Data are presented as mean ± SEM of three independent biological experiments (*n* = 3 membranes), each performed in technical triplicates (wells), which were averaged prior to analysis to generate a single value per biological replicate. * *p* < 0.05.

Mineralized matrix deposition and osteogenic activity of MG-63 cells exposed to membrane eluates were evaluated by Alizarin Red staining and alkaline phosphatase (ALP) activity. At day 1, calcium deposition was minimal and comparable among all groups. By day 7, Alb-PRF eluates induced a noticeable increase in mineralized nodules compared with the other groups, whereas Alb-nCHA-PRF and Alb-nCHA-PRF + DOX maintained lower levels. At day 21, the difference became more evident, with Alb-PRF supporting the highest calcium deposition, followed by intermediate levels in Alb-nCHA-PRF + DOX, and the lowest in Alb-nCHA-PRF (Figure 8).

**Figure 9:**
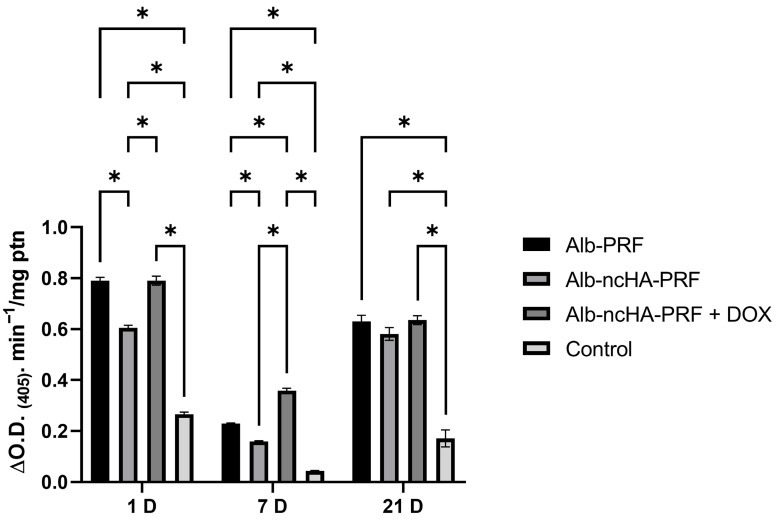
Alkaline phosphatase (ALP) activity (represented as the measured variation in optical density at 405 nm, normalized by protein content) in MG-63 cells exposed to eluates from Alb-PRF, Alb-nCHA-PRF, and Alb-nCHA-PRF+DOX membranes at 1, 7, and 21 days. Data are presented as mean ± SEM of three independent biological experiments (*n* = 3 membranes), each performed in technical triplicates (wells), which were averaged prior to analysis to generate a single value per biological replicate. * *p* < 0.05.

ALP activity followed a complementary pattern. At day 1, no significant differences were observed between groups. At day 7, a peak of enzyme activity was detected in the Alb-nCHA-PRF + DOX group, which was significantly higher than both Alb-PRF and Alb-nCHA-PRF. By day 21, ALP levels increased in all conditions, consistent with the progression from early differentiation to late mineralization (Figure 9).

### 2.9. Secretome Analysis

**Figure 10:**
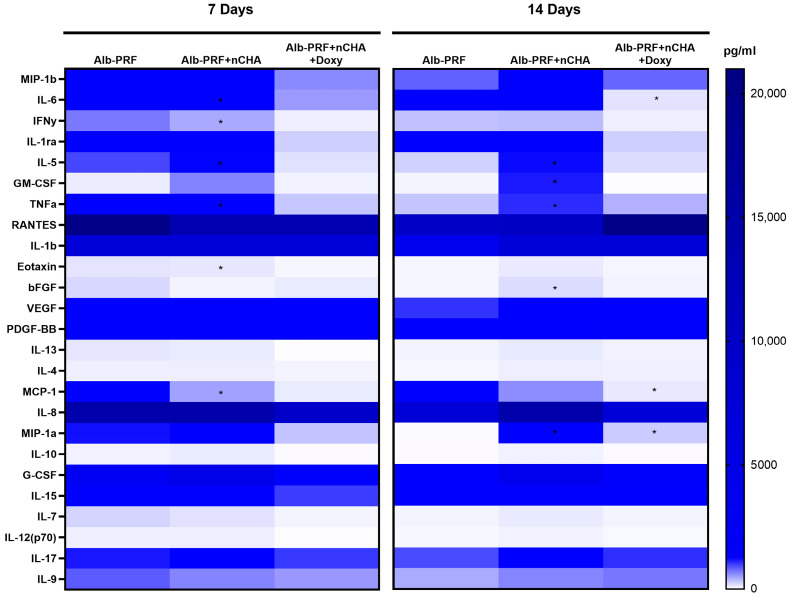
Heatmap representation of cytokine and growth factor release from Alb-PRF, Alb-nCHA-PRF, and Alb-nCHA-PRF + DOX membranes after 7 and 14 days of incubation. Data are expressed as mean concentrations (pg/mL). Warmer colors represent higher levels, while cooler colors indicate reduced secretion. Data represents the mean of four independent biological replicates (*n* = 4 membranes), each analyzed in triplicate technical replicates (wells), which were averaged prior to analysis to generate a single value per biological replicate. An asterisk indicates statistical significance compared with other groups at the same experimental time (*p* < 0.05).

The release of cytokines and growth factors from Alb-PRF, Alb-nCHA-PRF, and Alb-nCHA-PRF + DOX membranes was evaluated by multiplex immunoassay after 7 and 14 days of incubation. Heatmap representation of the 27 analytes revealed distinct secretion profiles among the groups (Figure 10).

At 7 days, Alb-PRF membranes released higher levels of several cytokines and growth factors, including IL-6, IL-1ra, MCP-1, IL-8, G-CSF, and VEGF. Alb-nCHA-PRF membranes showed a global reduction in these factors, with lower concentrations of IL-1ra, MCP-1, and G-CSF, while modest increases in GM-CSF and bFGF were observed. Incorporation of doxycycline resulted in even more pronounced downregulation of the secretome, with marked decreases in IL-6, IFN-γ, IL-5, MCP-1, and G-CSF, although some factors such as RANTES, VEGF, and PDGF-BB remained detectable at comparable levels to the other groups.

At 14 days, the overall release of cytokines declined in all groups. Alb-PRF still maintained measurable levels of several analytes, whereas Alb-nCHA-PRF secretions remained reduced. Alb-nCHA-PRF + DOX membranes continued to exhibit a downregulated profile, with particularly low levels of IL-6, IL-1ra, and MCP-1, while factors like VEGF and PDGF-BB persisted at moderate levels. These results demonstrate that the addition of nCHA reduces the release of multiple cytokines and growth factors from Alb-PRF membranes, and that incorporation of doxycycline further attenuates this profile, suggesting a modulatory effect of the antibiotic on the regenerative secretome. All the complete results, with means, SD and *p*-values, are described in Supplementary Table S2 (for 7 days) and Table S3 (for 14 days).

To verify whether the reduced secretome observed in nCHA-containing membranes could result from adsorption of soluble cytokines by the nanostructured spheres, an additional control experiment was performed using Alb-PRF-conditioned medium incubated for 24 h with sterile nCHA microspheres at the same mass as used in the composite formulations. Cytokine and growth factor concentrations were remeasured using the same 27-plex immunoassay panel. The results (Table S1) showed selective adsorption of several analytes, including MIP-1β, IL-1β, IL-6, G-CSF, and bFGF, while key mediators such as VEGF and PDGF-BB remained largely unchanged. These data indicate that nCHA can partially sequester certain cytokines from PRF eluates, consistent with the reduced secretome profile observed in the composite membranes, but without eliminating critical angiogenic and osteogenic factors.

## 3. Discussion

The present proof-of-concept study was designed to evaluate the feasibility of combining albumin-PRF, nanostructured carbonated hydroxyapatite (nCHA), and doxycycline into a single multifunctional membrane capable of supporting bone regeneration while providing local antimicrobial protection. This composite strategy aims to integrate the biological activity of PRF with the osteoconductive properties of nCHA and the bacteriostatic action of doxycycline, addressing two key postoperative challenges—early infection control and sustained regenerative signaling. The results therefore provide feasibility data to guide further optimization and in vivo validation.

Our study demonstrated that albumin-PRF membranes can be successfully functionalized with nanostructured carbonated hydroxyapatite and doxycycline, generating a composite with dual activity. The 4.25 mg/membrane doxycycline loading used in this study was previously optimized through preliminary release testing (Supplementary Figure S1) and validated in vivo in a rat bone defect model [6]. This concentration provided sustained local levels sufficient for antimicrobial activity without reaching cytotoxic thresholds for osteoblastic cells. The incorporation of doxycycline into the mineral phase was evidenced by autofluorescence, which demonstrated preferential retention within the nCHA microspheres even after 14 days of incubation. This observation is consistent with the well-established affinity of tetracyclines for calcium ions and apatite surfaces, a property that has long been exploited for the detection of mineralized tissues [19], and more recently revisited for drug delivery applications [20]. Nanostructured hydroxyapatite is known to provide a high surface area and abundant ionic binding sites, which facilitate drug adsorption and gradual release [21]. In our system, this mechanism was reflected in the release profile, which showed limited variation up to 96 h followed by a marked increase at 168 h, suggesting strong initial binding followed by progressive desorption as ionic equilibrium was established.

The antimicrobial assays demonstrated that eluates from Alb-nCHA-PRF + DOX inhibited the growth of both *Enterococcus faecalis* and *Staphylococcus aureus* at the 1/256 dilution after 1 and 7 days. However, the quantitative CFU analysis revealed distinct responses between the two pathogens: *E. faecalis* exhibited a profile consistent with a sustained bacteriostatic effect, whereas *S. aureus* showed complete bacterial eradication within the detection limits of the assay. This differential behavior is consistent with the known susceptibility profiles of these Gram-positive species, since *S. aureus* is typically more sensitive to doxycycline than *E. faecalis*, which possesses intrinsic mechanisms of reduced permeability and active efflux [22].

The ability of the hybrid membrane to maintain inhibitory or bactericidal concentrations for at least one week suggests that doxycycline was released locally at therapeutically relevant levels. Such localized delivery may be clinically advantageous, as sustained antibiotic exposure at the graft interface can prevent early contamination and improve regenerative outcomes while minimizing systemic exposure [23,24]. The strong inhibition observed against *S. aureus* is particularly relevant from a translational standpoint, since this pathogen is the primary cause of osteomyelitis, post-grafting infections, and implant-associated bone loss. Nevertheless, the coexistence of bacteriostatic outcomes with *E. faecalis* reinforces the need for dose optimization. A proportion of doxycycline is likely to remain adsorbed onto the nCHA surface, which could restrict its long-term diffusion while contributing to the sustained bacteriostatic profile observed. This behavior aligns with previous reports showing that calcium phosphate-bound doxycycline releases gradually and maintains sub-micromolar concentrations in bone tissue for several days after local delivery. Such levels are sufficient to inhibit common Gram-positive pathogens without reaching cytotoxic peaks that might interfere with osteoblast activity. However, prolonged subinhibitory exposure may favor the emergence of resistant strains [25]; therefore, future work should define release kinetics ensuring effective but safe local concentrations and expand testing to multispecies biofilm models that better reflect the clinical infection environment.

Recent experimental studies with doxycycline-functionalized biomaterials demonstrated positive effects on bone repair and local infection control [26], reinforcing the potential of this strategy. Similar antibacterial activity has been reported in doxycycline-doped polymeric membranes, which promoted osteoblast growth and immunomodulatory responses in vitro [18], and in doxycycline-functionalized bone substitutes that enhanced bone formation in vivo [17], further reinforcing that the mineral component not only serves as an effective carrier enabling controlled local drug release but also as a modulator of the regenerative microenvironment. Complementing these findings, a hydroxyapatite–doxycycline (HADOX) system in a rat model at high risk of MRONJ increased newly formed bone versus HA alone and reduced early inflammatory responses after dental extraction, supporting the translational relevance of local DOX delivery via mineral carriers [20]. Taken together, these studies reinforce that the mineral component not only serves as an effective carrier enabling controlled local drug release but also as a modulator of the regenerative microenvironment.

In the present study, cytocompatibility was confirmed through an ISO 10993-5:2009; Biological evaluation of medical devices—Part 5: Tests for in vitro cytotoxicity. International Organization for Standardization: Geneva, Switzerland, 2009-compliant extract assay, in which all membrane types preserved MG-63 viability above the 70% cutoff across all extract concentrations and exposure times. Importantly, the cytotoxicity workflow followed internationally recognized standards for 2D static assays, including validated mass-to-volume extraction ratios and exposure conditions, ensuring that the evaluation of membrane biocompatibility was conducted within established methodological boundaries. These findings were further supported by a multiparametric viability panel (XTT, NR uptake, and CVDE), which independently demonstrated preserved mitochondrial function, lysosomal integrity, and cell density after exposure to 100% extracts. Together, these data establish that the Alb-PRF-based composites are cytocompatible under standardized conditions and provide a reliable biological baseline within the limitations of in vitro assessments.

In vitro and in vivo studies have reported that doxycycline-functionalized scaffolds stimulate osteoblastic proliferation and differentiation, and the expression of osteogenic markers such as alkaline phosphatase (ALP) and bone morphogenetic proteins [17,18]. The exposure of osteoblast-like cells to the membranes elicited distinct osteogenic effects: Alb-PRF eluates favored higher proliferation (days 3–5) and greater late mineralization (day 21), whereas adding nCHA reduced such effects; notably, the presence of doxycycline partially rescued proliferation and produced a clear peak in ALP at day 7—an early differentiation signal. This pattern is coherent with the known biology of PRF, which provides sustained release of pro-regenerative mediators (e.g., PDGF, TGF-β, VEGF) capable of supporting cell proliferation and migration as well as osteogenic maturation over time [4,27]. The variability we observed after integrating a mineral phase is also consistent with recent evidence that PRF–graft composites can alter the biological “milieu” depending on preparation, where the calcium phosphate microspheres may adsorb and reduce the initial biological availability of molecular mediators, impacting cell behavior [13].

The early ALP rise with doxycycline aligns with studies showing that DOX-functionalized barrier materials can promote osteoblast proliferation/differentiation and immunomodulatory readouts, although magnitude and direction are dose- and context-dependent [17,18]. Mechanistically, our secretome data (robust secretion in Alb-PRF; attenuation with nCHA; further down-modulation with DOX, with selective persistence of important growth factors such as VEGF/PDGF-BB) offer a plausible link between formulation and phenotype: reduced MCP-1/G-CSF/IL-6 may limit recruitment and proliferative cues, while preserved angiogenic signals could support later tissue integration even under a dampened inflammatory background. These differences reinforce that the “ALP-early/mineral-late” pattern [28] observed here is biologically plausible, although it remains model-dependent. These findings may suggest a trade-off depending on the choice of combination: Alb-PRF maximizes trophic signals for proliferation/mineralization, while nCHA + DOX tends to anticipate differentiation events (ALP) under a more restrained secretome; fine-tuning of doxycycline dose and release kinetics, together with tests in more complex cellular and in vivo models, will be necessary to clarify the translational balance of these effects.

This interpretation must be considered within the limitations of the MG-63 model, which is widely used in biomaterial research but differs from primary human osteoblasts in terms of maturation dynamics and marker expression [29]. The use of the MG-63 osteoblast-like cell line in the present work was intentional and grounded in standardization and comparability. MG-63 cells are one of the most extensively employed models in biomaterial and bone tissue engineering research, providing a reproducible and well-characterized platform for the early evaluation of cytocompatibility, differentiation, and mineralization potential. Although immortalized, MG-63 cells retain key osteoblastic features, including alkaline phosphatase activity, collagen synthesis, and calcium nodule formation when exposed to osteogenic stimuli, according to the results in numerous previous studies investigating platelet concentrates [6]. These properties make them a reliable first-line model for screening novel bone regenerative systems before validation in primary human osteoblasts or mesenchymal stem cells.

The quantitative Live/Dead analysis clarified that the decrease in green fluorescence observed in the Alb-nCHA-PRF and Alb-nCHA-PRF + DOX membranes does not necessarily indicate cytotoxicity. These images capture the population of autologous blood cells originally trapped within the PRF matrix, which undergo programmed apoptosis as part of the natural degradation and remodeling process. The presence of mineral microspheres and sustained doxycycline release may accentuate this decline by altering diffusion dynamics and transiently increasing local drug concentrations, but such effects are expected and transient. Importantly, the independent MG-63 assays confirmed normal metabolic activity and proliferation under standard exposure conditions, supporting the overall biocompatibility of the hybrid membranes.

The regenerative effects of platelet concentrates are largely attributed to their ability to release cytokines and growth factors that regulate cell proliferation, migration, angiogenesis, and differentiation [4,27]. PRF matrices sustain the availability of VEGF, PDGF, TGF-β and other mediators for several days, creating a paracrine environment favorable to osteogenesis and tissue repair [30]. In the present study, we observed that Alb-PRF maintained the most robust secretome, while the presence of nCHA reduced the release of several mediators, and doxycycline further downregulated this profile. The reduction in cytokine release observed in the nCHA-containing membranes prompted an additional adsorption control experiment to investigate whether this effect could result from sequestration of soluble mediators by the mineral phase. The results (Table S1) confirmed selective adsorption of several proinflammatory cytokines, while key pro-angiogenic mediators such as VEGF and PDGF-BB remained largely unaffected. These findings indicate that nCHA does not globally deplete the PRF secretome but rather acts as a transient reservoir for specific molecules, consistent with previous reports on calcium phosphate interactions with plasma proteins [14].

In this context, the combined effects of nCHA and doxycycline should be interpreted as modulation of the biological milieu rather than suppression of regenerative signaling. Indeed, the Alb-nCHA-PRF + DOX group displayed the highest ALP activity at day 7—suggesting an early differentiation stimulus—and maintained intermediate calcium deposition at day 21, higher than nCHA alone though slightly lower than Alb-PRF. This pattern suggests a temporal shift in osteogenic events, likely reflecting both transient cytokine adsorption by nCHA and the antiproteolytic action of doxycycline, which together reshape, rather than impair, the osteogenic trajectory of the hybrid membrane.

The progressive reduction in viability observed among the blood-derived cells entrapped within the PRF-based membranes, particularly in the presence of doxycycline, likely reflects a physiological turnover rather than a deleterious effect. These cells are known to act transiently as sources of growth factors, cytokines, and matrix proteins that drive the early stages of tissue regeneration. Their gradual apoptosis and resorption are inherent to PRF remodeling and coincide with the diffusion and consumption of their bioactive products. The more pronounced decline observed in the Alb-nCHA-PRF + DOX group may indicate accelerated release dynamics modulated by the antibiotic and by interactions between the fibrin–albumin matrix and the mineral phase. Importantly, this temporal loss of viability does not imply loss of function; rather, it suggests that the entrapped cells had already discharged a substantial fraction of their regenerative secretome, consistent with the maintained mineralization activity and the selective cytokine adsorption pattern demonstrated in the adsorption control experiment (Table S1).

The additional modulation induced by doxycycline appears to complement the selective adsorption pattern observed for nCHA. Beyond its well-established antimicrobial role, doxycycline exerts antiproteolytic and anti-inflammatory effects through matrix metalloproteinase (MMP) inhibition and modulation of cytokine processing [31]. These actions may transiently dampen inflammatory mediators while preserving trophic and angiogenic cues within the microenvironment. Consistently, VEGF and PDGF-BB remained detectable across all experimental groups, and their persistence is mechanistically relevant: VEGF orchestrates the coupling of angiogenesis and bone formation, whereas PDGF-BB recruits progenitor cells and supports early matrix organization [32]. Thus, the combined influence of nCHA and DOX does not imply a loss of regenerative signaling, but rather a recalibration of the cytokine balance—favoring early differentiation events (as reflected by the ALP peak) while maintaining the molecular support required for subsequent tissue integration.

The structural persistence of the fibrin–albumin matrix is a critical factor for barrier function and scaffold performance in bone regeneration. In vivo studies have shown that Alb-PRF exhibits significantly greater resistance to degradation compared with standard PRF, remaining intact for several weeks, whereas conventional PRF membranes are rapidly resorbed [8,9,10]. This extended stability, together with prolonged release of bioactive mediators, makes Alb-PRF an attractive option in procedures that require sustained barrier function. In our evaluation, Alb-PRF and Alb-nCHA-PRF membranes preserved their integrity up to 14 days in culture, with only minor compaction of the fibrin–albumin matrix. By contrast, Alb-nCHA-PRF + DOX membranes displayed greater fragility, with progressive detachment of nCHA microspheres after 7 days. This instability may reflect chemical interactions between doxycycline, calcium ions, and fibrin, potentially weakening the cohesion of the composite. Similar effects have been reported in other composite formulations where excessive mineral phase or chemical modifications altered the mechanical integrity of PRF-based scaffolds [13]. These observations highlight the importance of fine-tuning the formulation. In the present study, we adopted the Alb-gel/PRF ratio described in the earliest protocols, but it is conceivable that increasing the relative proportion of albumin gel in DOX-containing groups could mitigate fragility and improve stability. Future studies should therefore explore how adjusting the Alb-gel/PRF balance, in combination with controlled nCHA and DOX loading, may preserve membrane integrity while retaining antimicrobial and regenerative functionality. It should be emphasized, however, that these observations were based solely on macroscopic and photographic assessments. No quantitative mechanical or degradation tests (e.g., tensile strength or mass loss) were performed, and therefore the reported stability should be regarded as a qualitative indicator of morphological preservation. Future work will incorporate standardized mechanical and degradation assays to better characterize the structural behavior of these membranes under physiological conditions.

Our results align with reports that albumin-enriched PRF displays superior stability compared with conventional PRF, sustaining bioactivity over longer periods [8,9]. Incorporating nCHA improved handling, in line with “sticky bone” concepts used clinically, while the biological readouts varied with formulation, in accordance with results from clinical trials and in vitro studies [13,14]. By integrating nCHA and doxycycline, the membranes demonstrated the potential to combine structural reinforcement with local antimicrobial protection, an attractive feature in clinical scenarios where contamination is a major risk—such as peri-implant defects, bone augmentation in previously infected sites, or revision surgeries. The preferential retention of doxycycline within nCHA microspheres and its sustained release at bacteriostatic levels suggest that this composite may offer a valuable adjunct to conventional regenerative strategies by delivering local protection while minimizing systemic antibiotic exposure. At the same time, the data reveal formulation-dependent trade-offs: while Alb-PRF alone favored robust trophic support for proliferation and mineralization, the addition of nCHA and especially doxycycline altered the secretome and shifted osteogenic responses toward early differentiation at the expense of late mineralization. These findings underscore the importance of optimizing the balance between antimicrobial efficacy and regenerative signaling. Future studies should therefore refine the Alb-gel/PRF ratio, adjust the nCHA content, and titrate doxycycline doses to maximize stability and bioactivity.

Nevertheless, this study has some limitations that should be acknowledged when interpreting the findings. First, the experiments were performed under in vitro conditions using MG-63 osteoblast-like cells, which, although widely adopted in biomaterial research, do not fully replicate the complexity of primary human osteoblasts or the in vivo bone environment, and should be confirmed with human osteoblastic or mesenchymal stem cell cultures and in vivo models. The mineralization profile should address osteogenic gene expression (*RUNX2*, *COL1A1*, *OCN*) in future studies to help correlate early transcriptional events with the mineralization and secretome patterns observed here. Likewise, antimicrobial testing was restricted to single-species suspension culture and thus does not capture the dynamics of polymicrobial biofilms commonly encountered in oral and orthopedic infections. It should also be noted that the incorporation of doxycycline required the addition of 1 mL of aqueous solution to the Alb-nCHA-PRF mixture, introducing a modest (~11%) dilution of the liquid phase. Although no differences in gelation behavior or gross morphology were observed, minor effects on the concentration of soluble mediators cannot be excluded. Future studies will include volume-matched pilot controls and basic physicochemical measurements (pH and ionic strength) to better isolate potential solvent-related influences while maintaining clinical relevance. On the other hand, these limitations do not diminish the value of the study as an exploratory step in understanding how Alb-PRF, nCHA, and doxycycline interact within a composite system. By combining complementary methodologies—including fluorescence-based drug localization, quantitative release profiling, antimicrobial assays, cytocompatibility testing, osteogenic readouts, and secretome analysis—the results provided a preliminary picture of both the strengths and trade-offs of this strategy, and highlight opportunities for future research, particularly studies that explore different doxycycline doses and release kinetics, incorporate polymicrobial biofilm models, and validate the findings in vivo. Finally, this work represents a preliminary but essential phase in the translational pathway of Alb-PRF-based composites. By integrating structural, antimicrobial, and biological data, it establishes the mechanistic and safety rationale necessary to guide subsequent in vivo validation. Conducting this proof-of-concept stage prior to animal experimentation aligns with the principles of the 3Rs—Replacement, Reduction, and Refinement—ensuring that future studies are ethically justified, quantitatively informed, and scientifically optimized.

Future studies should move beyond single-species assays and osteoblast-like monocultures to models that better reproduce the clinical environment, including polymicrobial biofilms, co-cultures with immune and osteogenic cells, and systems that evaluate angiogenesis. Ultimately, validation in critical-size defect models will be necessary to establish clinical relevance. Nevertheless, the present preliminary findings suggest that Alb-PRF is a versatile autologous matrix that can be functionalized with both inorganic phases and bioactive agents. With careful optimization, Alb-PRF + nCHA + DOX composites may represent a promising next-generation scaffold able to meet the dual challenges of infection control and bone regeneration.

## 4. Materials and Methods

### 4.1. Synthesis and Characterization of Nanostructured Carbonated Hydroxyapatite (nCHA)

CHA powder was synthesized at the Bioceramics Laboratory of the Brazilian Center for Physics Research (CBPF, Rio de Janeiro, Brazil) using a wet precipitation method. Briefly, an aqueous solution of dibasic ammonium phosphate was added dropwise to a calcium nitrate solution at 37 °C, under constant stirring, with pH adjusted to 12. The resulting precipitate was dried in an oven and sieved to a maximum particle size of 250 µm. The carbonate substitution level was approximately 6%. nCHA microspheres were produced by mixing the powder with sodium alginate solution (6:1 ratio, *w*/*w*) and dripping the suspension into 0.3 M calcium chloride solution using a 26G needle. The spheres were allowed to crosslink for 24 h at 4 °C, washed, and dried at 60 °C. Fractions between 420 and 600 µm were selected using a precision sieve (Bertel Metalurgica Ltd., Sao Paulo, Brazil), packed in 1 g aliquots, and sterilized by ethylene oxide.

Physicochemical characterization was performed by Fourier transform infrared spectroscopy (FTIR, IRPrestige-21, Shimadzu, Kyoto, Japan), X-ray diffraction (XRD, X’Pert, PANalytical, Almelo, Netherlands) and dynamic light scattering (DLS, Litesizer 500, Anton Paar, Graz, Austria equipment model), confirming carbonate incorporation, low crystallinity, and nanoscale particle size, and reported in a previous study [21]. Briefly, XRD confirmed the characteristic reflections of stoichiometric hydroxyapatite (Ca/P = 1.66) without secondary phases, and FTIR spectra showed typical OH^−^ and PO_4_^3−^ bands along with carbonate substitutions around 1450 cm^−1^, consistent with a nanostructured carbonated apatite. SEM revealed spherical granules with a porous nanostructured surface morphology favorable for drug adsorption. The nCHA exhibited low crystallinity with nanocrystallite sizes in the 20–60 nm range, typical of biomimetic carbonated apatites.

### 4.2. Preparation of Alb-PRF, Alb-nCHA-PRF and Alb-nCHA-PRF + DOX Membranes

The study was approved by the Ethics Committee of the Hospital Universitário Antônio Pedro (protocol 3.432.068), and all procedures followed the Declaration of Helsinki. Peripheral blood was collected from four healthy donors (28–35 years, both sexes) without recent use of anticoagulant or antimicrobial medication. Venipuncture was performed using 21G butterfly needles into 9 mL additive-free tubes (Vacutube Seco, Biocon^®^, São Paulo, Brazil).

Alb-PRF membranes were obtained according to [9]. After centrifugation (700 RCF-max, 8 min, horizontal rotor centrifuge BIO-PRF, Jupiter, FL, USA), the platelet-poor plasma fraction was heat-denatured in an APAG device (Silfradent, Santa Sofia, Italy) for 10 min at 75 °C to form albumin gel. Once cooled to ~37 °C, 1.5 mL of Alb-gel was mixed with 2.5 mL of liquid PRF in 6-well plates inclined at 45°, leading to polymerization of stable Alb-PRF membranes. For Alb-nCHA-PRF membranes, 1 g of sterile nCHA microspheres was added before mixing the Alb-gel and liquid PRF. For Alb-nCHA-PRF + DOX, the nCHA microspheres were pre-incubated with 1 mL of freshly prepared doxycycline solution (4.25 mg/mL; Merck, Darmstadt, Germany) for 5–15 min prior to polymerization, yielding a final drug content of 4.25 mg per membrane. No equivalent solvent volume was added to the Alb-PRF or Alb-nCHA-PRF controls, as the aim was to reproduce the final formulations as they were clinically prepared and applied, rather than to create volume-matched experimental variants. The apparent gelation time and macroscopic handling characteristics were comparable among groups. Polymerization occurred within 5–10 min, and the membranes were maintained in DMEM (Dulbecco’s Modified Eagle Medium, Gibco, São Paulo, Brazil) at 37 °C, 5% CO_2_ until testing.

The morphological integrity of Alb-PRF, Alb-nCHA-PRF, and Alb-nCHA-PRF + DOX membranes was assessed during incubation in DMEM at 37 °C for up to 14 days. Membranes were maintained individually in 6-well plates, and their macroscopic integrity was monitored at 1, 7, and 14 days by digital photography under standardized conditions. Changes in shape, thickness, and overall preservation of the fibrin–albumin structure were qualitatively documented.

### 4.3. Incorporation of Doxycycline

Prior to selecting the final doxycycline concentration, a preliminary dose–response study was conducted using nCHA microspheres loaded with different DOX contents (1.06, 2.12, 4.25, 8.5, and 17 mg per batch). The release profiles indicated that higher loads (≥8.5 mg) caused a rapid burst release exceeding concentrations known to be cytotoxic to osteoblastic cells, whereas lower doses (≤2.12 mg) failed to reach the minimal inhibitory concentration (MIC_90_) for *S. aureus* and *E. faecalis*. The 4.25 mg dose yielded the most balanced release pattern, achieving sustained levels within the antimicrobial but non-cytotoxic range. These data are presented in Supplementary Figure S2.

Doxycycline hyclate powder (Merck, Darmstadt, Germany) was dissolved in deionized water to obtain a sterile solution at 4.25 mg/mL, filtered through a 0.22 µm membrane, and protected from light until use. For the Alb-nCHA-PRF + DOX group, 1 g of nCHA microspheres was incubated with 1 mL of the doxycycline solution for 5–15 min prior to incorporation into the albumin/PRF mixture, resulting in a final drug content of 4.25 mg per membrane.

The ability of Alb-nCHA-PRF + DOX membranes to act as a local drug delivery system was investigated by monitoring doxycycline release over time. After polymerization, each membrane was placed in a dialysis bag (12 kDa cutoff, Sigma-Aldrich, São Paulo, Brazil) containing 4 mL of sterile PBS (pH 7.4) and incubated at 37 °C under humidified conditions. At predetermined intervals (1, 24, 48, 72, 96, and 168 h), 200 µL of the eluate was collected and immediately replaced with an equal volume of fresh PBS to maintain sink conditions. Doxycycline concentration was quantified spectrophotometrically at 275 nm (NanoDrop™ 2000, Thermo Fisher Scientific, Waltham, MA, USA). Calibration was performed with freshly prepared DOX standards (0.01–0.50 mg/mL), and the linearity of the calibration curve (R^2^ > 0.99) was confirmed prior to sample analysis. Blank correction using PBS was applied to all measurements. A standard calibration curve was constructed with serial dilutions of doxycycline to convert absorbance values into absolute drug concentrations. The results were expressed both as the cumulative release (µg/mL) and as the percentage of the total doxycycline initially incorporated into the membranes.

### 4.4. Live/Dead Staining of Blood Cells Within Alb-PRF-Based Membranes

Cell viability within the membranes was assessed immediately after polymerization and after incubation in DMEM for 1 and 7 days. Membranes (Alb-PRF, Alb-nCHA-PRF, and Alb-nCHA-PRF + DOX) were gently rinsed in PBS and transferred to glass-bottom dishes. A Live/Dead working solution containing calcein-AM (2 µM) and ethidium homodimer-1 (EthD-1, 4 µM) in PBS was added to fully cover each membrane, followed by incubation for 30 min at 37 °C, and protected from light. Samples were then washed twice with PBS and imaged by fluorescence microscopy (FITC channel for calcein-AM; TRITC/Cy3 channel for EthD-1). Images were acquired using identical exposure, gain, and magnification settings across all groups.

Quantitative analysis of viability was performed using Image-Pro Plus software (version 6.0, Media Cybernetics, Rockville, MD, USA). For each sample, five non-overlapping representative fields were analyzed. The total areas occupied by green (viable) and red (non-viable) fluorescence were segmented using color thresholding and measured in pixels. The ratio of live to total fluorescence area was calculated for each field and normalized to the colonizable membrane surface, excluding regions occupied by mineral microspheres. The data are expressed as mean ± SEM of three independent donors.

### 4.5. Secretome Analysis

The release of cytokines and growth factors from Alb-PRF, Alb-nCHA-PRF, and Alb-nCHA-PRF + DOX membranes was quantified in conditioned media collected after 7 and 14 days of incubation. Immediately after polymerization, each membrane was incubated in 4 mL of antibiotic-free DMEM at 37 °C and 5% CO_2_. At each experimental time point, the conditioned media were collected, centrifuged to remove cell debris, and stored at −80 °C until analysis. Cytokine and growth factor concentrations were measured using a commercial multiplex immunoassay kit (Bio-Plex Pro™ Human Cytokine 27-plex Assay, Bio-Rad, Hercules, CA, USA), based on magnetic bead technology (xMAP, Luminex Corp., Austin, TX, USA). The panel included IL-1β, IL-1RA, IL-2, IL-4, IL-5, IL-6, IL-7, IL-8, IL-9, IL-10, IL-12(p70), IL-13, IL-15, IL-17, CCL11 (eotaxin), FGF-basic, G-CSF, GM-CSF, IFN-γ, CXCL10 (IP-10), CCL2 (MCP-1), CCL3 (MIP-1α), CCL4 (MIP-1β), PDGF-BB, CCL5 (RANTES), TNF-α, and VEGF. The assays were performed on a Bio-Plex MAGPIX™ System (Bio-Rad) following the manufacturer’s instructions, and analyte concentrations were calculated using Bio-Plex Manager software (v.3.0). The results are expressed in pg/mL and were normalized to the membrane volume used for conditioning. Each experimental group was tested in biological triplicates.

To evaluate whether the reduced secretome observed in nCHA-containing membranes could be influenced by cytokine adsorption, an additional control experiment was performed. Conditioned medium obtained from Alb-PRF membranes after 7 days of incubation was collected, centrifuged to remove debris, and incubated for 24 h at 37 °C with sterile nCHA microspheres (1 g per 4 mL medium), corresponding to the same mineral mass used in the composite formulations. After incubation, the suspensions were centrifuged, and the supernatants were collected and analyzed using the same multiplex immunoassay panel described above. Cytokine concentrations were compared with those from Alb-PRF-conditioned medium incubated without nCHA under identical conditions. Each condition was tested in biological triplicates, and the results were analyzed for significant differences using two-tailed *t*-tests with false discovery rate (FDR) correction. The results are presented in the Supplementary Materials (Table S1).

### 4.6. Antimicrobial Activity

The antimicrobial activity of the membranes was evaluated by determining the minimum inhibitory concentration (MIC) against *Enterococcus faecalis* (ATCC 29212, American Type Culture Collection, Manassas, VA, USA) or *Staphylococcus aureus* (ATCC 25923, American Type Culture Collection, Manassas, VA, USA), using the broth microdilution method according to the CLSI guidelines (2012). Bacterial suspensions were prepared in tryptic soy broth (TSB) and adjusted to the 0.5 McFarland standard (~1.5 × 10^8^ CFU/mL). Eluates obtained from Alb-PRF, Alb-nCHA-PRF, and Alb-nCHA-PRF + DOX membranes after 1 and 7 days of culture were serially diluted two-fold in 96-well plates, starting from the 100% conditioned medium. Each well contained 990 µL of diluted eluate inoculated with 10 µL of bacterial suspension, and plates were incubated at 37 °C for 18 h. MIC was defined as the lowest eluate dilution that prevented visible turbidity. Negative controls (sterile TSB and PBS) and positive bacterial growth controls were included in all assays.

To distinguish bacteriostatic from bactericidal effects, aliquots from MIC wells were streaked onto blood agar and incubated for 24 h, after which colony forming units were counted with the help of the Image Pro Plus software. All experiments were performed in biological triplicates.

### 4.7. Cytocompatibility Assays

MG-63 human osteoblast-like cells (ATCC^®^ CRL-1427™) between passages 8 and 12 were used for all experiments. Cells were cultured in Dulbecco’s Modified Eagle Medium (DMEM, Gibco, São Paulo, Brazil) supplemented with 10% fetal bovine serum (FBS) and 1% penicillin/streptomycin and maintained at 37 °C with 5% CO_2_.

Cytocompatibility was assessed using an extract-based assay based on the principles of the ISO 10993-5:2009 guidelines. Membrane extracts were prepared according to ISO 10993-12:2021: 2021; Biological evaluation of medical devices—Part 12: Sample preparation and reference materials. International Organization for Standardization: Geneva, Switzerland, 2021, by incubating each membrane (average mass of 3.2 g) in Dulbecco’s Modified Eagle Medium (DMEM, serum-free, antibiotic-free) at a ratio of 200 mg/mL for 24 h at 37 °C under sterile conditions. This preparation was defined as the 100% extract, and two additional dilutions (50% and 25%) were prepared using fresh DMEM as the diluent.

MG-63 cells were seeded in 96-well plates at 5 × 10^3^ cells/well and allowed to adhere for 24 h, resulting in a consistent sub-confluent monolayer appropriate for extract-based cytotoxicity testing. Cells were then exposed to 100%, 50%, or 25% extract concentrations for 24, 48, or 72 h, maintaining identical culture conditions for all time points. A medium-only group served as the experimental control, while high-density polystyrene beads (2 mg/mL) were employed as the negative control (C−), and a latex extract (2 mg/mL) served as the positive control (C+), both prepared according to the ISO guidelines. After each exposure period, cell viability was quantified using an MTT assay. Briefly, cells were incubated with MTT solution (0.5 mg/mL in DMEM) for 3 h at 37 °C, followed by dissolution of formazan crystals in DMSO and spectrophotometric reading at 570 nm measured using a Synergy II microplate reader (BioTek Instruments, Winooski, VT, USA). Viability was expressed as a percentage of the negative control, and values ≥70% were interpreted as non-cytotoxic in accordance with ISO 10993-5. All experiments were performed in triplicate.

For the confirmation of cytocompatibility, a multiparametric assay was performed using a commercial kit (In Cytotox, Xenometrix, Allschwil, Switzerland). Membranes were incubated in 4 mL antibiotic-free DMEM for 24 h at 37 °C, and the resulting conditioned media were diluted to a proportion corresponding to 200 mg/mL of material/media (100% ISO 10993-12 extract). These conditioned media were applied to MG-63 cells seeded in 96-well plates at a density of 2.0 × 10^4^ cells/well. Cell viability was assessed by a multiparametric approach combining XTT reduction to evaluate mitochondrial metabolic activity, neutral red (NR) uptake to assess lysosomal function, and crystal violet dye exclusion (CVDE) to estimate cell number through DNA content. Positive controls for cytotoxicity consisted of latex extract (200 mg/mL), while unconditioned media served as the negative control. Optical density was measured using a Synergy II microplate reader (BioTek Instruments, Winooski, VT, USA). Each experiment was performed with five technical replicates and three biological replicates pooled for analysis.

### 4.8. Cell Proliferation

The effect of the membranes on osteoblast proliferation was evaluated using conditioned media obtained after 24 h of incubation of Alb-PRF, Alb-nCHA-PRF, and Alb-nCHA-PRF + DOX membranes. Human MG-63 osteoblast-like cells were seeded in 96-well plates at a density of 1.0 × 10^3^ cells per well and treated with 150 µL of conditioned medium diluted to 25% in DMEM supplemented with 5% FBS. Fresh conditioned media were replaced every 72 h to maintain a constant exposure throughout the experiment. Cell proliferation was assessed at 1, 3, 5, and 7 days using the crystal violet dye exclusion (CVDE) assay. After each time point, the cells were washed with PBS, fixed, and stained with crystal violet solution, followed by multiple washes and solubilization in 10% acetic acid. Absorbance was measured at 540 nm in a Synergy II microplate reader (BioTek Instruments, Winooski, VT, USA), and the results are expressed as a percentage relative to the untreated controls. Each experimental group was analyzed in five technical replicates and pooled biological triplicates.

### 4.9. Biomineralization

The osteogenic potential of the membranes was investigated by evaluating both calcium deposition and alkaline phosphatase (ALP) activity in human MG-63 cells exposed to conditioned media. Cells were seeded in 48-well plates at a density of 2.0 × 10^4^ cells per well and, after 24 h, the culture medium was replaced with 25% conditioned medium supplemented with 5% FBS. Media were partially renewed three times per week by replacing half the volume with fresh conditioned medium under the same conditions, with full changes of culture media at 24 h before each collection of the supernatant (at days 6, 13 and 20). Calcium deposition in the extracellular matrix was quantified at 1, 7, and 21 days using alizarin red staining. At each time point, cells were fixed with 4% paraformaldehyde, stained with alizarin red solution, and subsequently washed to remove excess dye. Mineralized nodules were visualized microscopically, and the bound dye was eluted for spectrophotometric quantification. In parallel, ALP activity was evaluated as an early marker of osteogenic differentiation. Supernatants collected at 1, 7, and 21 days were incubated with p-nitrophenyl phosphate (pNPP) substrate, and the reaction product p-nitrophenol was quantified spectrophotometrically at 405 nm in a Sinergy II microplate reader (Biotek Instruments, Winooski, VT, USA). Enzyme activity was normalized to total protein content, determined by the Bradford assay, to account for variations in cell density among samples. The results are expressed as units of ALP activity per mg of total protein. All experiments were performed in biological triplicates, with technical triplicates for each condition.

### 4.10. Statistical Analysis

All quantitative data were expressed as mean ± standard error of the mean (SEM) from independent biological replicates. Each biological replicate corresponded to an independent experiment (independently prepared membranes from different donors), typically *n* = 3 unless otherwise specified. Technical replicates (wells or repeated measurements) were averaged and considered as a single value for each biological replicate, which was used as the statistical unit. The exact number and nature of biological and technical replicates for each experiment are indicated in the corresponding figure legends. Due to the limited sample size and the non-normal distribution of some variables, non-parametric statistical tests were applied. Comparisons among groups were performed using the Kruskal–Wallis test, followed by Dunn’s multiple comparisons post hoc test when appropriate. Analyses were conducted using GraphPad Prism software (v.9.0, GraphPad Software Inc., San Diego, CA, USA). A value of *p* < 0.05 was considered statistically significant. For multiplex cytokine data (Luminex analysis), *p*-values were adjusted for multiple comparisons using the Benjamini–Hochberg false discovery rate (FDR) method, as recommended for high-dimensional datasets. A corrected q-value < 0.05 was considered statistically significant.

## 5. Conclusions

Alb-PRF membranes provided a stable autologous scaffold capable of sustaining cell viability and releasing regenerative cytokines and growth factors. Incorporation of nCHA introduced a mineral component that altered the secretome and attenuated some proliferative and mineralization responses, while the addition of doxycycline conferred sustained bacteriostatic activity against *E. faecalis* and promoted early osteogenic differentiation signals. These preliminary findings suggest that Alb-PRF can be further engineered as a dual-function platform for bone regeneration, combining antimicrobial protection with regenerative potential, although formulation-dependent trade-offs highlight the need for dose optimization and validation in more complex models.

## Figures and Tables

**Table 1:** Doxycycline release over time (instantaneous vs. cumulative).

Time Point	Mean Conc. ± SEM (µg/mL)	Mass in Media at Time (mg)	Cumulative Mass Released (mg) ^1^	Cumulative % of Total (4.25 mg)
24 h	41.49 ± 5.818	0.166	0.166	3.91%
48 h	39.99 ± 1.866	0.160	0.168	3.96%
72 h	55.93 ± 8.271	0.224	0.240	5.65%
96 h	47.00 ± 9.964	0.188	0.215	5.06%
168 h	82.07 ± 8.457 *	0.328	0.364	8.57%

Data are presented as mean ± SEM of four independent experiments (*n* = 4), with measurements obtained from independent membranes. ^1^ Cumulative mass at time t_n_ = 4·C_n4_·C_n4_·C_n_ + 0.2·(C_1_ + C_2_ +…+ C_n−1_)0.2·(C_1_ + C_2_ +…+ C_n−1_)0.2·(C_1_ + C_2_ +…+ C_n−1_), where concentrations in µg/mL were converted to mg. An asterisk indicates significative statistical difference from other experimental times (*p* < 0.05).

## Data Availability

The original contributions presented in this study are included in the article/Supplementary Material. Further inquiries can be directed to the corresponding author(s).

## Supplementary Materials

The following supporting information can be downloaded at: https://www.mdpi.com/article/10.3390/ijms27083639/s1.
