# Supplementary material for: Alb-PRF Hybrid Membranes Functionalized with Carbonated Hydroxyapatite and Doxycycline for Bone Regeneration and Antimicrobial Control: An In Vitro Study
Source: Int J Mol Sci. 2026 Apr 19;27(8):3639. doi: 10.3390/ijms27083639 (PMC13115618; doi:10.3390/ijms27083639)
Supplement: Supplementary file 1 [file ijms-27-03639-s001.zip › ijms-3874636-supplementary.pdf]

Table S1. Cytokine entrapment of cytokines on nCHA spheres after exposure to Alb-PRF eluates

Analyte	Alb-PRF extract (mean ± SD)	Alb-PRF extract + carbo spheres (mean ± SD)	p-value	Adjusted p (FDR)
MIP-1b	78.83 ± 6.59	28.08 ± 1.29	0.0043	**0.0148***
IL-6	22.55 ± 1.64	32.49 ± 1.30	0.0015	**0.0110***
IFNy	1.84 ± 0.14	2.73 ± 0.17	0.0028	**0.0110***
IL-1ra	933.42 ± 61.53	1166.45 ± 110.32	0.0466	0.1016
IL-5	5.37 ± 0.56	1.97 ± 3.42	0.2254	0.3182
GM-CSF	0.14 ± 0.25	0.00 ± 0.00	0.4226	0.4820
TNFa	32.46 ± 5.00	27.44 ± 4.23	0.2573	0.3250
RANTES	10386.83 ± 7538.19	1272.21 ± 63.75	0.1712	0.2740
IL-1b	2.30 ± 0.15	1.34 ± 0.18	0.0025	**0.0110***
Eotaxin	5.55 ± 0.76	1.83 ± 0.17	0.0107	0.0320
bFGF	1.47 ± 0.04	1.26 ± 0.03	0.0019	**0.0110***
MCP-1	7.26 ± 3.08	4.30 ± 1.82	0.2406	0.3208
PDGF-BB	141.89 ± 23.29	133.98 ± 10.57	0.6321	0.6321
IP-10	178.21 ± 27.04	45.38 ± 2.29	0.0131	**0.0348***
IL-13	0.36 ± 0.25	0.23 ± 0.07	0.4418	0.4820
IL-4	0.22 ± 0.07	0.26 ± 0.00	0.4226	0.4820
VEGF	285.78 ± 3.99	274.59 ± 5.73	0.0570	0.1140
IL-8	663.99 ± 42.57	715.57 ± 111.22	0.5157	0.5382
MIP-1a	36.75 ± 3.22	16.41 ± 2.78	0.0013	**0.0110***
IL-10	1.04 ± 0.13	0.50 ± 0.49	0.1937	0.2905
G-CSF	41.76 ± 2.57	6.15 ± 0.86	0.0007	0.0110
IL-15	28.37 ± 13.71	54.66 ± 14.84	0.0876	0.1617
IL-17	0.85 ± 0.56	0.00 ± 0.00	0.1179	0.2021
IL-9	39.47 ± 4.20	29.31 ± 1.38	0.0417	0.1001

An asterisk indicates statistically significant differences between groups after false discovery rate (FDR) correction (adjusted $p < 0.05$).

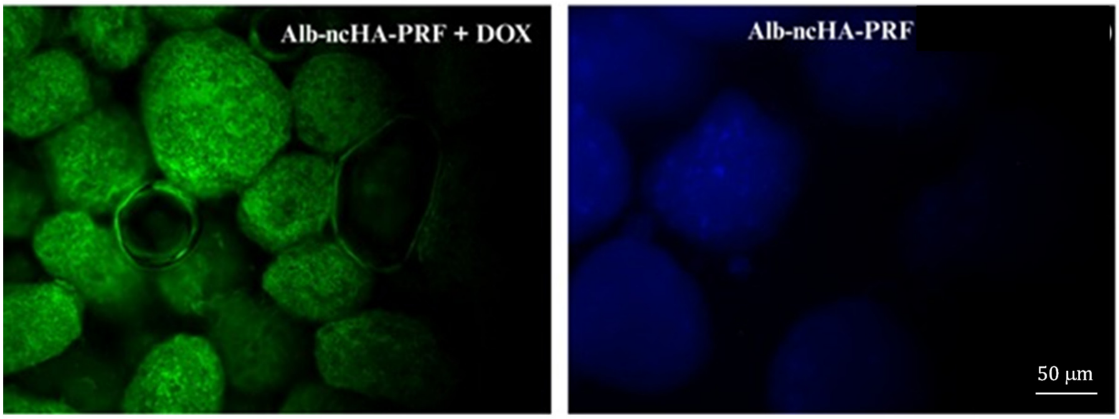

Figure S1. Fluorescence microscopy of Alb-nCHA-PRF + DOX membranes after 14 days of incubation. The doxycycline signal (green) remained concentrated within the mineral microspheres, confirming its preferential retention in the nCHA phase. No fluorescence was detected in the Alb-nCHA-PRF membranes without doxycycline (negative control, right panel). Scale bar = 50 μm.

Table S2: Cytokine and growth factor release from the membranes after 7 days of incubation.

Analyte	Alb-PRF (mean ± SD)	Alb-PRF + nCHA (mean ± SD)	Alb-PRF + nCHA + DOX (mean ± SD)	p-ANOVA	p-FDR	Significance
**MIP-1b**	341.88 ± 324.45	415.85 ± 167.11	46.29 ± 13.52	0.155	0.237	—
**IL-6**	463.73 ± 119.42	772.30 ± 334.90	39.88 ± 53.46	**0.014**	**0.048**	*
**IFN-γ**	52.99 ± 26.87	34.14 ± 19.75	5.95 ± 5.18	**0.016**	**0.048**	*
**IL-1ra**	431.76 ± 389.31	306.39 ± 328.61	19.24 ± 8.24	0.287	0.365	—
**IL-5**	71.41 ± 46.95	98.31 ± 38.04	12.24 ± 3.26	0.058	0.091	—
**GM-CSF**	7.42 ± 6.10	48.82 ± 27.32	5.07 ± 4.11	**0.029**	**0.042**	*
**TNF-α**	155.13 ± 176.05	105.13 ± 18.35	22.57 ± 5.27	0.343	0.418	—
**RANTES**	20258.00 ± 0.00	13574.38 ± 11576.38	13604.45 ± 11524.29	0.630	0.691	—
**IL-1β**	6949.00 ± 0.00	6949.00 ± 0.00	6949.00 ± 0.00	—	—	—
**Eotaxin**	10.73 ± 4.56	9.92 ± 2.69	3.36 ± 0.71	**0.049**	**0.049**	*
**bFGF**	15.96 ± 3.89	4.42 ± 1.43	7.99 ± 3.81	**0.012**	**0.055**	**--**
**VEGF**	142.80 ± 17.40	179.72 ± 14.83	121.18 ± 50.28	0.155	0.237	—
**PDGF-BB**	310.89 ± 132.71	388.88 ± 288.97	343.05 ± 263.54	0.923	0.954	—
**IL-13**	10.01 ± 2.54	8.61 ± 2.98	0.68 ± 0.55	**0.005**	**0.029**	*
**IL-4**	6.38 ± 0.54	6.97 ± 0.23	3.95 ± 3.42	0.227	0.311	—
**MCP-1**	417.43 ± 378.08	37.28 ± 15.06	8.60 ± 4.52	**0.011**	**0.017**	*
**IL-8**	14486.00 ± 0.00	14486.00 ± 0.00	9678.40 ± 8327.00	0.422	0.493	—
**MIP-1α**	91.09 ± 134.70	284.54 ± 69.59	23.75 ± 22.51	0.027	0.060	—
**IL-10**	5.22 ± 4.30	8.45 ± 5.75	1.33 ± 0.00	0.190	0.277	—
**G-CSF**	2262.18 ± 1618.21	4254.26 ± 1545.49	1155.32 ± 222.20	0.067	0.099	—
**IL-15**	333.10 ± 59.86	506.58 ± 106.60	75.39 ± 102.41	**0.0036**	**0.026**	*
**IL-7**	17.01 ± 5.57	11.50 ± 10.47	4.15 ± 0.00	0.149	0.225	—
**IL-12**	6.57 ± 2.63	7.02 ± 6.55	0.88 ± 0.20	**0.020**	**0.027**	*
**IL-17**	88.31 ± 11.56	100.47 ± 1.40	76.46 ± 9.23	0.039	0.072	—
**IL-9**	64.06 ± 3.37	48.56 ± 10.49	40.65 ± 12.16	0.058	0.091	—

Table S3: Cytokine and growth factor release from the membranes after 14 days of incubation.

Analyte	Alb-PRF (mean ± SD)	Alb-PRF + nCHA (mean ± SD)	Alb-PRF + nCHA + DOX (mean ± SD)	p-ANOVA	p-FDR	Sig
**MIP-1b**	61.87 ± 86.91	382.96 ± 95.18	59.65 ± 11.58	0.035	0.222	—
**IL-6**	161.68 ± 228.09	634.72 ± 266.44	11.36 ± 12.47	**0.0107**	**0.042**	*
**IFN-γ**	24.59 ± 32.39	26.50 ± 13.63	6.31 ± 2.16	0.603	0.677	—
**IL-1ra**	233.97 ± 325.15	155.71 ± 144.18	19.33 ± 2.93	0.622	0.677	—
**IL-5**	18.05 ± 19.71	93.07 ± 54.95	13.77 ± 2.45	**0.016**	**0.041**	*
**GM-CSF**	4.26 ± 5.42	87.94 ± 78.70	0.43 ± 0.00	**0.024**	**0.040**	*
**TNF-α**	23.24 ± 31.87	81.22 ± 33.10	30.61 ± 6.20	**0.020**	**0.047**	*
**RANTES**	10129.62 ± 14323.69	10217.73 ± 14199.09	20258.00 ± 0.00	0.650	0.677	—
**IL-1β**	3474.59 ± 4913.56	6949.00 ± 0.00	6949.00 ± 0.00	0.465	0.677	—
**Eotaxin**	3.35 ± 4.47	8.90 ± 3.18	3.77 ± 0.77	0.298	0.617	—
**bFGF**	3.37 ± 1.06	13.83 ± 3.42	4.32 ± 0.94	**0.028**	**0.028**	*
**VEGF**	78.92 ± 95.74	189.64 ± 7.37	136.95 ± 0.90	0.281	0.617	—
**PDGF-BB**	567.83 ± 798.62	294.14 ± 361.56	1063.75 ± 702.61	0.554	0.677	—
**IL-13**	4.56 ± 5.92	8.99 ± 4.30	5.47 ± 0.67	0.600	0.677	—
**IL-4**	2.90 ± 4.06	6.88 ± 0.24	5.87 ± 0.00	0.346	0.617	—
**MCP-1**	573.75 ± 802.20	45.06 ± 24.49	9.37 ± 2.27	**0.485**	**0.495**	*
**IL-8**	7243.44 ± 10242.52	14486.00 ± 0.00	7275.24 ± 10197.55	0.650	0.677	—
**MIP-1α**	1.52 ± 1.29	302.37 ± 4.34	20.56 ± 0.06	**0.000002**	**0.000061**	*
**IL-10**	1.33 ± 0.00	4.92 ± 4.07	1.28 ± 0.07	0.339	0.617	—
**G-CSF**	408.24 ± 568.32	3550.39 ± 1754.84	1199.36 ± 30.07	0.119	0.424	—
**IL-15**	107.82 ± 121.12	447.46 ± 53.72	102.00 ± 129.34	0.076	0.381	—
**IL-7**	4.16 ± 0.02	8.55 ± 6.18	4.16 ± 0.02	0.463	0.677	—
**IL-12**	3.00 ± 1.62	5.29 ± 3.35	2.60 ± 1.33	0.523	0.677	—
**IL-17**	70.33 ± 8.90	105.42 ± 2.85	80.20 ± 1.83	0.016	0.206	—
**IL-9**	33.77 ± 42.48	48.01 ± 16.50	53.89 ± 11.00	0.766	0.766	—

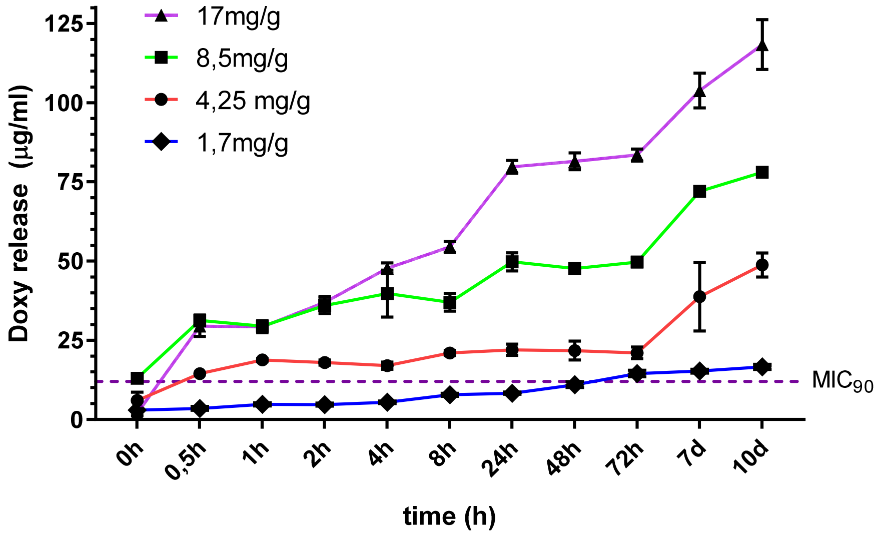

Figure S2. Preliminary doxycycline dose–response and release kinetics from nCHA microspheres. Cumulative DOX release (µg/mL) from nCHA microspheres loaded with 1.7, 4.25, 8.5, and 17 mg DOX per g of microspheres over 10 days. The dashed line indicates the MIC_{90} for *S. aureus*. Higher loads (≥ 8.5 mg) produced rapid burst release exceeding cytotoxic levels, whereas lower loads (≤ 1.7 mg) failed to reach the inhibitory range. The 4.25 mg condition provided the most balanced antimicrobial and cytocompatible profile.

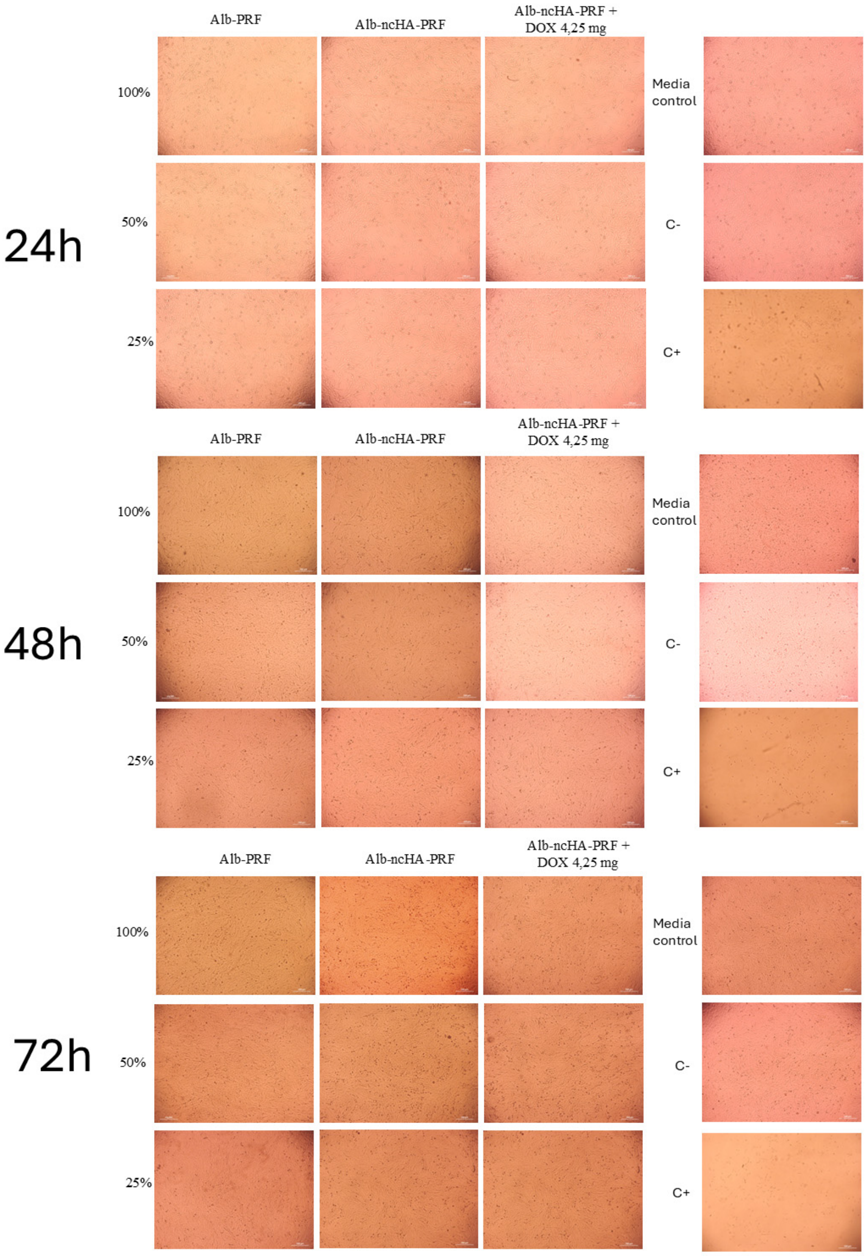

Figure S3. Representative optical micrographs of MG-63 osteoblast-like cells after 24 h, 48 h, and 72 h exposure to membrane extracts prepared according to ISO 10993-5 (200 mg/mL extraction; 100%, 50%, and 25% concentrations). Panels show formazan formation in cells treated with extracts from Alb-PRF, Alb-nCHA-PRF, and Alb-nCHA-PRF+DOX membranes, alongside the medium control, negative control (C–, polystyrene high density beads), and positive control (C+; latex extract). All images acquired under identical lighting and magnification settings (scale bar = 200 μm).
